# Supplementary material for: Modeling glioblastoma heterogeneity as a dynamic network of cell states
Source: Mol Syst Biol. 2021 Sep 16;17(9):e10105. doi: 10.15252/msb.202010105 (PMC8444284; doi:10.15252/msb.202010105)
Supplement: Supplementary file 6 — Source Data for Figure 5 [file MSB-17-e10105-s004.zip › Figure5A_sourcedata/GSEA_3071/nosigdb_stateIV.GseaPreranked.1622795164626/WANG_MESENCHYMAL_PMID_28697342.html]

Details for gene set WANG\_MESENCHYMAL\_PMID\_28697342[GSEA]

|  || Dataset | state53071 |
| Phenotype | NoPhenotypeAvailable |
| Upregulated in class | na\_neg |
| GeneSet | WANG\_MESENCHYMAL\_PMID\_28697342 |
| Enrichment Score (ES) | -0.752336 |
| Normalized Enrichment Score (NES) | -3.0532563 |
| Nominal p-value | 0.0 |
| FDR q-value | 0.0 |
| FWER p-Value | 0.0 |
Table: GSEA Results Summary

  

Fig 1: Enrichment plot: WANG\_MESENCHYMAL\_PMID\_28697342      
 Profile of the Running ES Score & Positions of GeneSet Members on the Rank Ordered List

  

| PROBE | GENE SYMBOL | GENE\_TITLE | RANK IN GENE LIST | RANK METRIC SCORE | RUNNING ES | CORE ENRICHMENT || 1 | COL1A2 |  |  | 137 | 1.425 | 0.0085 | No |
| 2 | SYNGR2 |  |  | 1577 | 0.371 | -0.2561 | No |
| 3 | BCL3 |  |  | 2125 | 0.280 | -0.3533 | No |
| 4 | BACE2 |  |  | 2695 | -0.294 | -0.4544 | No |
| 5 | ITGB1 |  |  | 2817 | -0.308 | -0.4699 | No |
| 6 | LY96 |  |  | 2935 | -0.323 | -0.4843 | No |
| 7 | GPRC5A |  |  | 3356 | -0.382 | -0.5549 | No |
| 8 | VDR |  |  | 3524 | -0.414 | -0.5766 | No |
| 9 | RAB27A |  |  | 3744 | -0.454 | -0.6073 | No |
| 10 | FTL |  |  | 4350 | -0.615 | -0.7074 | No |
| 11 | BNC2 |  |  | 4450 | -0.654 | -0.7104 | No |
| 12 | SDC1 |  |  | 4663 | -0.770 | -0.7320 | Yes |
| 13 | ARPC1B |  |  | 4771 | -0.840 | -0.7320 | Yes |
| 14 | CNN2 |  |  | 4774 | -0.842 | -0.7120 | Yes |
| 15 | CTSB |  |  | 4948 | -1.012 | -0.7203 | Yes |
| 16 | NNMT |  |  | 5039 | -1.168 | -0.7091 | Yes |
| 17 | GLIPR1 |  |  | 5096 | -1.279 | -0.6888 | Yes |
| 18 | S100A11 |  |  | 5100 | -1.292 | -0.6581 | Yes |
| 19 | TGFBI |  |  | 5104 | -1.305 | -0.6270 | Yes |
| 20 | P4HA2 |  |  | 5127 | -1.346 | -0.5986 | Yes |
| 21 | TNFAIP8 |  |  | 5130 | -1.363 | -0.5660 | Yes |
| 22 | LOX |  |  | 5149 | -1.418 | -0.5350 | Yes |
| 23 | PRSS23 |  |  | 5179 | -1.562 | -0.5027 | Yes |
| 24 | COL15A1 |  |  | 5208 | -1.691 | -0.4671 | Yes |
| 25 | PLAUR |  |  | 5213 | -1.721 | -0.4261 | Yes |
| 26 | THBS1 |  |  | 5222 | -1.800 | -0.3841 | Yes |
| 27 | LUM |  |  | 5271 | -2.487 | -0.3329 | Yes |
| 28 | COL5A1 |  |  | 5276 | -2.605 | -0.2706 | Yes |
| 29 | CAV1 |  |  | 5281 | -2.850 | -0.2023 | Yes |
| 30 | COL3A1 |  |  | 5287 | -3.508 | -0.1183 | Yes |
| 31 | COL1A1 |  |  | 5290 | -4.897 | 0.0000 | Yes |
Table: GSEA details [plain text format]

  

Fig 2: WANG\_MESENCHYMAL\_PMID\_28697342: Random ES distribution      
 Gene set null distribution of ES for **WANG\_MESENCHYMAL\_PMID\_28697342**
